# Editorial

**DOI:** 10.1365/s43439-020-00001-8

**Published:** 2020-09-18

**Authors:** Dennis-Kenji Kipker, Peter Pagel

**Affiliations:** 1 Universität Bremen, Bremen, Deutschland; 2 Wiesbaden, Deutschland

## Dear reader,

We are pleased to present the first issue of the new *International Cybersecurity Law Review* (*ICLR*). Especially in the present time it is once again more than clear: digitalisation is more important than ever, and sometimes we are forced to digitise, be it as a company or as a private person. This makes it more important than ever that the networked IT systems that form the basis of all digitalisation are secure. This applies on the one hand to the protection of stored personal data against unauthorised access and manipulation, in other words, data security, but also, quite apart from data protection, to ensuring the functionality of IT systems.

We are in an age of increasing risks of digitalisation in all areas of society, and legislators have recognised this—not only in Germany and the European Union, but worldwide: in hardly any other field there have been so many regulations in recent years as in cyber security. In Germany, one thinks of the IT Security Act and the IT Security Act 2.0, at the European level of the Network and Information Security Directive or the EU Cybersecurity Act, but also of the Federal Law for the Protection of Critical Information Infrastructures in the Russian Federation, the US Cybersecurity and Infrastructure Security Agency Act, or the Chinese Cybersecurity Law (CSL), which has attracted worldwide attention since 2016, as well as the recently enacted Chinese Cryptography Law, which places global data flows to and from China under completely new legal conditions.

In view of this multitude of regulations, some of which are so new that neither case law nor accompanying legal literature exists, it is all the more important to have a reliable source at hand that prepares the relevant information in a practical, up-to-date, comprehensible and first-hand manner. This is the task of the *ICLR*: companies, legislators, politicians, authorities and the scientific community are all equally addressed by this new format. The interdisciplinary nature of this topic, which is located at the interface between law and technology, requires that the explanations are presented in a comprehensible and appealing way not only for lawyers, but also for engineers, computer scientists and technicians.

The Springer *ICLR* wants to take on this important task and challenge in equal measure—so we are all pleased that this new journal has found its way to you, dear readers. In the future, the *ICLR* will cover all questions relating to international cybersecurity from a legal and technical perspective—whereby the legal perspective should always be the starting point for all further explanations. The formats that you will find in this journal range from classic specialist articles, guidelines, interviews, short reports, current developments and news, and reports on case law, to comparative law synopses. Contributions are printed both in German and, especially for international texts, in English. The *ICLR* will initially be published twice a year.

In this first double issue you can expect various current topics reflecting the multifaceted nature of international cyber security law: the new EU directives on digital content and the contracts for the sale of goods, legal issues of cross-border data transfer and the related risk assessment in China, an update on the framework conditions and the functioning of the instruments of the EU Cybersecurity Act, global disinformation campaigns and their social impact, politically and legally highly controversial responsibilities in Tanzanian cybersecurity law, the much-cited “Cybersecurity Review” in China, a presentation of the Chinese cryptography law, the (non-)implementation of the EU NIS Directive in the Member States, why the issue of cyber security is important, especially for children’s toys, on intergovernmental cyber sovereignty, the Budapest Convention on Cybercrime, data security in Croatia under conditions of remote work due to the corona virus, and data security and data protection management for video conferencing software.

We now wish you insightful and exciting reading on the digital topics that move the economy, research and society in our time. And if you have any suggestions or remarks, or perhaps would like to contribute to an interesting topic yourself, please feel free to contact us at any time!

Dr. Dennis-Kenji Kipker, Editor-in-Chief, Bremen, Germany

Peter Pagel, Springer Vieweg, Wiesbaden, Germany

## Liebe Leserin, lieber Leser,

wir freuen uns, Ihnen die erste Ausgabe der neuen Zeitschrift für Cybersicherheit und Recht (International Cybersecurity Law Review, kurz: ICLR) präsentieren zu können. Gerade in der jetzigen Zeit wird wieder einmal mehr als deutlich: Die Digitalisierung ist bedeutungsvoller denn je, und manchmal werden wir auch zur Digitalisierung gezwungen, sei es als Unternehmen oder als Privatperson. Umso wichtiger ist es, dass die vernetzten IT-Systeme, die die Grundlage einer jeden Digitalisierung bilden, sicher sind. Dies betrifft einerseits den Schutz von gespeicherten personenbezogenen Daten vor unbefugter Kenntnisnahme und Manipulation, also die Datensicherheit, andererseits aber auch, losgelöst vom Datenschutz, die Sicherstellung der Funktionsfähigkeit von IT-Systemen.

Wir befinden uns in einem Zeitalter zunehmender Risiken der Digitalisierung aller Gesellschaftsbereiche. Dies haben auch die Gesetzgeber erkannt – nicht nur in Deutschland und der Europäischen Union, sondern weltweit: In kaum einem anderen Feld hat es in den letzten Jahren so viel Regulierung gegeben wie in der Cybersicherheit. Man denke in Deutschland an das IT-Sicherheitsgesetz und das IT-Sicherheitsgesetz 2.0, auf europäischer Ebene an die Richtlinie zur Netz- und Informationssicherheit oder an den EU Cybersecurity Act, aber auch an das Bundesgesetz zum Schutz kritischer Informationsinfrastrukturen in der Russischen Föderation, den US-amerikanischen Cybersecurity and Infrastructure Security Agency Act, oder an das Chinese Cybersecurity Law (CSL), das seit dem Jahr 2016 weltweite Beachtung fand, sowie das jüngst in Kraft getretene Chinese Cryptography Law, das die globalen Datenströme von und nach China unter völlig neue rechtliche Bedingungen stellt.

Im Angesicht dieser Vielzahl von Regelungen, die teils so neu sind, dass weder Rechtsprechung noch begleitende juristische Literatur existieren, ist es umso wichtiger, eine verlässliche Quelle zur Hand zu haben, die die relevanten Informationen praxisgerecht, aktuell, verständlich und aus erster Hand aufbereitet. Dies ist die Aufgabe des ICLR: Unternehmen, Gesetzgeber, Politik, Behörden und die Wissenschaft werden durch dieses neue Format gleichermaßen angesprochen. Dabei gebietet es die Interdisziplinarität dieses an der Schnittstelle von Recht und Technik liegenden Themas, die Ausführungen nicht nur für Juristen, sondern auch für Ingenieure, Informatiker und Techniker verständlich und ansprechend aufbereitet darzustellen.

Der Springer ICLR will sich dieser wichtigen Aufgabe und Herausforderung gleichermaßen annehmen – umso mehr freut es uns, dass diese neue Zeitschrift den Weg zu Ihnen, liebe Leserschaft, gefunden hat. Der ICLR wird künftig alle Fragen rund um die internationale Cybersecurity in rechtlicher und technischer Hinsicht abdecken –, wobei die juristische Betrachtung stets der Ausgangspunkt für alle weiteren Ausführungen sein soll. Die Formate, die Sie in dieser Zeitschrift finden werden, reichen von klassischen Fachbeiträgen über Leitfäden, Interviews, Kurzberichte, aktuellen Entwicklungen und Nachrichten sowie Rechtsprechungsreporten bis hin zu rechtsvergleichenden Synopsen – stets am Fachpublikum ausgerichtet. Beiträge werden sowohl in deutscher als auch – insbesondere für internationale Texte – in englischer Sprache abgedruckt. Der Erscheinungstermin des ICLR ist zunächst zweimal jährlich.

In dieser ersten Doppelausgabe erwarten Sie verschiedene aktuelle Themen, die den Facettenreichtum des internationalen Rechts der Cybersicherheit widerspiegeln: die neuen EU-Richtlinien über digitale Inhalte und den Warenkauf, Rechtsfragen des grenzüberschreitenden Datentransfers und die damit verbundene Risikobewertung in China, ein Update zu den Rahmenbedingungen und der Funktionsweise der Instrumente im EU Cybersecurity Act, globale Desinformationskampagnen und deren gesellschaftliche Auswirkungen, politisch und rechtlich hoch umstrittene Verantwortlichkeiten im tansanischen Cybersicherheitsrecht, der viel zitierte „Cybersecurity Review“ in China, eine Vorstellung des chinesischen Kryptografiegesetzes, die (Nicht‑)Umsetzung der EU NIS-Richtlinie in den Mitgliedstaaten, warum das Thema Cybersicherheit insbesondere auch für Kinderspielzeug wichtig ist, zur zwischenstaatlichen Cyber-Souveränität, zur Budapest-Konvention zur Cyber-Kriminalität, zur Datensicherheit in Kroatien unter den Bedingungen der Telearbeit aufgrund des Corona-Virus, und zum Datensicherheits- und Datenschutzmanagement für Videokonferenzsysteme.

Wir wünschen Ihnen nun eine erkenntnisreiche und spannende Lektüre zu den digitalen Themen, die Wirtschaft, Forschung und Gesellschaft in unserer Zeit bewegen. Und falls Sie Anregungen und Hinweise haben sollten oder vielleicht auch selbst gerne zu einem interessanten Thema einen Beitrag beisteuern möchten, so zögern Sie nicht, uns jederzeit zu kontaktieren!

Dr. Dennis-Kenji Kipker, Herausgeber, Bremen, Deutschland

Peter Pagel, Springer Vieweg, Wiesbaden, Deutschland

